# Tailored Physical Therapy in a Case of Tenotomy Post Hip Flexion Deformity With Structural Scoliosis: A Case Report

**DOI:** 10.7759/cureus.52276

**Published:** 2024-01-14

**Authors:** Ritika S Bhagwani, Pratik Phansopkar, Medhavi V Jagzape, Samruddhi M Karanjkar, Roshni R Nandanwar

**Affiliations:** 1 Musculoskeletal Physiotherapy, Ravi Nair Physiotherapy College, Datta Meghe Institute of Higher Education and Research, Wardha, IND

**Keywords:** physiotherapy, tumor, deformity, gait, contracture

## Abstract

Retroperitoneal tumours, mainly classified as malignant and benign, have a rare incidence. It includes major parts of the peritoneum, and surgical excision remains the optimal pathway to remove the tumour. As with any surgery, tumour resection comes with its own complications. These complications would manifest differently depending on patient adaptation or compensation for these drawbacks. Physiotherapy and its positive effects as a need after any surgical procedure become a boon when implemented as and when required. The present study describes the case of a 32-year-old woman who has complained of pain in her right hip for one year, along with forward-bending walking and difficulty sitting. The patient had a history of retroperitoneal tumour excision, after which, to compensate for the pain, she started walking by bending forward, which developed into a hip flexion deformity. The patient was managed by tenotomy and was referred to a physiotherapy outpatient (OPD) for further management. Our aim was to improve overall mobility through gait training and prevent relapses of the contracture.

## Introduction

A category of cancers known collectively as retroperitoneal tumours develop in the anatomical region known as the retroperitoneal space. The ipsilateral colon, mesocolon, pancreas, liver, and other vital organs are covalently bound to the retroperitoneal cavity. Retroperitoneal tumours, once considered a rare disorder, have been documented frequently in recent years; hence, they can no longer be classified as such. They can be categorised as benign or malignant tumours, single or numerous, solid, cystic, or both, and of various histologic kinds [1]. Retroperitoneal tumours (RPTs) originally develop from soft tissues, including fats, muscles, nerves, lymph nodes, and blood or lymphatic vessels. RPTs sometimes involve retroperitoneal organs, such as the kidneys, adrenal glands, pancreas, and intrapelvic organs (the bladder, uterus, ovaries, prostate, etc.). Invasive RPTs also involve major retroperitoneal lumen structures, such as the abdominal aorta, inferior vena cava, and ureters [2]. Spindle cell neoplasm, characterised histologically by a mixture of fat cells and fibroblast-like spindle cells in a matrix of collagen and mucoid material, is rare, and its incidence is low. It can occur in human soft tissue, bone, or in any part of the human body, such as the retroperitoneal space. Most of them occur at the young age of 20-40 years, occasionally appear in children, and their incidence has no significant differences between males and females [3]. Spindle cell tumours are rare soft tissue neoplasms with a low potential for malignancy. Atypical spindle cell tumours frequently occur in the limbs and limb girdles [4]. The posterior margins of these tumors frequently overlap the psoas muscle and retroperitoneal fat, making attaining negative margins difficult without visibility and precise dissection [5].

The mainstay of management for these tumours is surgical excision. Psoas major and minor muscles are often adherent to retroperitoneal tumors, and compartmental as well as radical resections performed involve the removal of the tumour along with some healthy tissue, including the psoas major muscle, to prevent the risk of recurrence. Post-surgical complications of hip flexion deformity result from a lack of physiotherapy rehabilitation, leading to muscle weakness and abnormal gait deviations caused by contracture formation. Improper gait adaptations alter the biomechanics of the spine and create deformities such as lumbar hyperlordosis, scoliosis, pelvic anteversion, etc. The current study presents the case of a 32-year-old female who came to the hospital with complaints of pain in her right hip for one year, which was gradual and progressive in nature. She underwent surgical management for a retroperitoneal tumour three years ago, and due to pain and discomfort, she started walking with forward bending deformity and developed flexion contracture at the hip, secondary to which she developed scoliosis. The patient was managed for deformity correction via tenotomy of the sartorius and was further managed by physiotherapy interventions.

## Case presentation

Patient information

A 32-year-old female came to the orthopaedic department of a tertiary care rural hospital with complaints of pain in the right hip associated with forward bending while walking for eight months. The patient was previously diagnosed with retroperitoneal mass for which she underwent explorative procedures, and was specifically diagnosed with spindle cell neoplasm in 2019. She was surgically operated on for tumour excision in 2019 and was simultaneously diagnosed with deep vein thrombosis in the right lower limb, which was further managed by inferior vena cava (IVC) filter placement. Post-operatively, due to complaints of severe pain, the patient reported that she started walking by bending forward and flexing her hips while walking or climbing the stairs. She also faced difficulty sitting down. Eventually, she started experiencing discomfort around her spine. The patient came to our hospital on December 15, 2022, and upon examination, she was diagnosed with fixed flexion contracture deformity of the right hip (50 degree deformity). Upon X-ray investigations, findings revealed scoliosis of the spine and an X-ray of the hip revealed flexion contracture of the right hip. The patient was surgically managed via tenotomy and was post-operatively referred to the physiotherapy outpatient department (OPD) for further management.

Clinical findings

An informed consent was obtained from the patient, which was done prior to the physical examination. On inspection, the patient was found in a supine lying position and was cooperative, with orientation to time, place, and person. The patient was subjected to skeletal traction over the right proximal tibia post-tenotomy. Upon side lying, right-sided scoliosis was observed. The right shoulder was found to be elevated, the interscapular distance from the spine was unequal, and the waistline was higher on the left side. On palpation, tenderness grade II was present over the right hip; squaring of both anterior superior iliac spines (ASIS) was absent. Tightness was present in the left trapezius, latissimus dorsi, and rhomboids.

Range of Motion and Manual Muscle Testing

**Figure 1:**
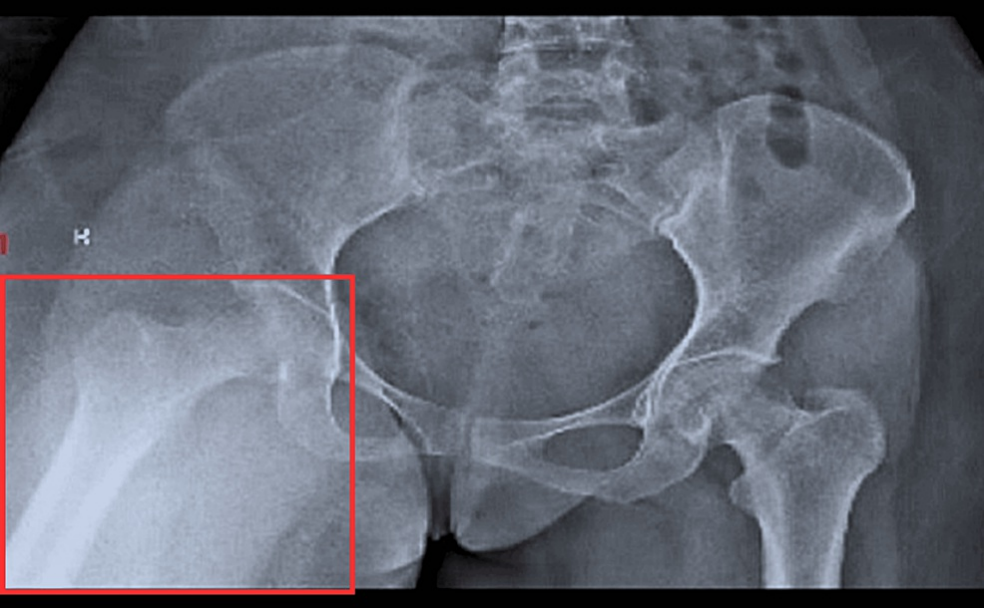
Pre-operative X-ray of right-sided hip The red square shows a fixed flexion deformity of 50^o^.

An examination was done after the removal of the traction. Pre-operative findings revealed a right-sided fixed hip flexion deformity of 50 degrees (Figure 1). Range of motion and manual muscle testing were used as outcome measures (Tables 1, 2). On examination, it was concluded that the right hip has an overall weakness of 3/5 with a restricted range of motion due to deformity.

**Table 1:** Range of motion for hip, knee and ankle joints post-operatively.

Range of Motion	Right side (in degree)	Left side (in degree)
Hip Flexion	0-55	0-90
Hip Extension	55-0	90-0
Hip Abduction	0-20	0-40
Hip Adduction	20-0	40-0
Knee Flexion	0-40	0-100
Knee Extension	40-0	100-0

**Table 2:** Manual muscle testing for joints of bilateral lower limbs.

Joint	Right	Left
Hip flexion	3/5	4/5
Hip extension	3/5	4/5
Knee flexion	3/5	4/5
Knee extension	3/5	4/5
Ankle plantarflexion	4/5	5/5
Ankle dorsiflexion	4/5	5/5

Physiotherapy management

**Figure 2:**
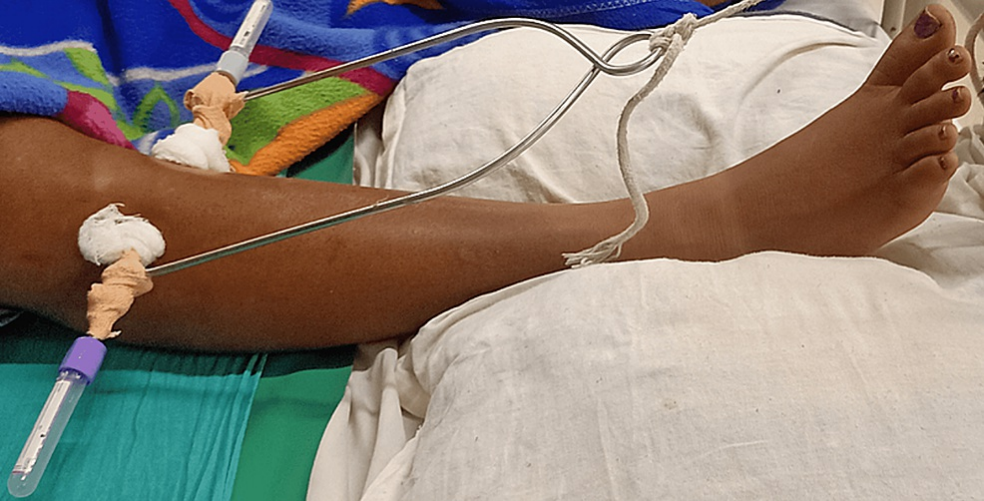
Proximal attachment of skeletal traction

**Figure 3:**
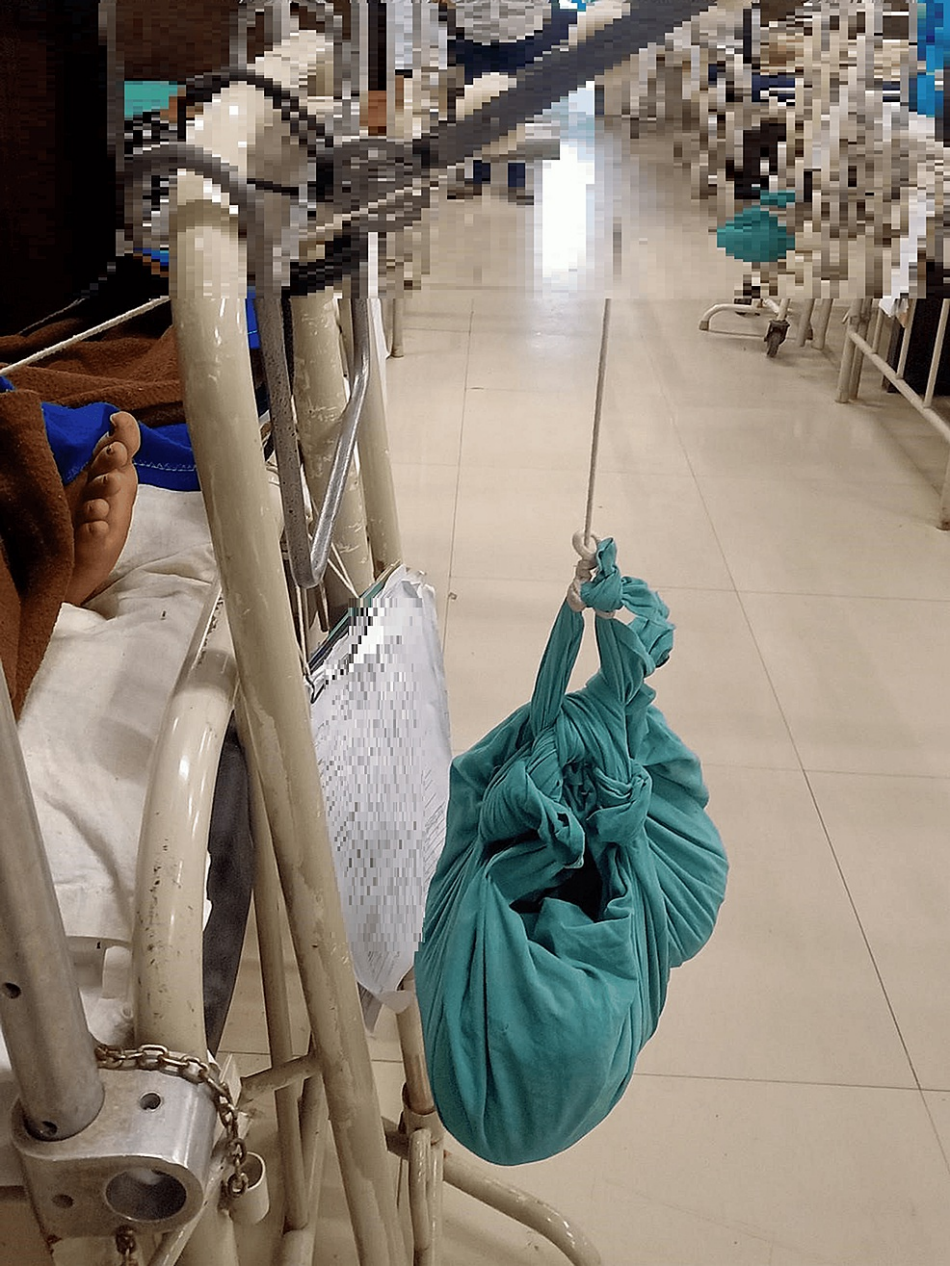
Weight attached to proximal tibial traction

Physiotherapy protocol is given below in the Table 3. Figures 2, 3 summarize the intervention given to the patient.

**Table 3:** Physiotherapy rehabilitation over the course of two months

Sr.no	Goals	Intervention	Repetitions
1.	Patient counselling	Educating patients about post-operative goals of physiotherapy and bed mobility precautions.	Before the beginning of the physiotherapy session.
2.	Postural correction	After removing the traction, the patient was turned to a side-lying position on the left side and placed a roll of pillow or foam under the curve to align the spine. The curve was corrected.	10 minutes, thrice each day, for a week.
3.	Improved respiratory capacity	Thoracic expansion Inspiratory muscle training device (IMT): Deep breathing exercises	10 reps, three times each day. Once a day, for a week 10 reps, thrice a day, for a week.
4.	Improve range of motion	Active range of motion within pain free range initially for hip flexion after removing the traction, progressing to wall slides. Knee range of motion through heel slides and dynamic quads.	10 reps for each range, twice a day for a week.
5.	Muscle Strengthening	Rhomboids strengthening of right side: 1. Prone lateral raise- Patient lies in prone position at the edge of bed on the right side. Patient was instructed to hold half kg water bottle and lift. 2. Wall slides: Patient is in standing position facing the wall, shoulders abducted, elbows flexed to 90 and extend elbows and shoulders parallel to the wall. Latissimus Dorsi strengthening of right side: Using therabands to perform pull downs, pull backs and adduction range. Lower limb strengthening: Beginning with static strengthening of quadriceps and hamstrings, progressing to weight cuffs of progressive weights for strength training. Initiated in gravity eliminated position, progressing to against gravity.	Each exercise beginning with 5 sec hold, progressing to 10 sec hold, twice a day.
6.	Muscle stretching	Stretching of back extensors on left side of the curve Latissimus dorsi stretching by lateral bending. Rhomboid stretching by scapular squeeze and cross arm stretch. Trapezius stretching by cervical lateral flexion and cervical flexion. Lateral trunk flexors stretching using therapy ball in side-lying.	Each stretch hold for 10 seconds, thrice a day for a week.
7.	Prevent recurrence of contracture	Sartorius active range of motion exercises (in pain free range)	4-5 times each day.
8.	Gait training	Non weight bearing ambulation beginning at 2 weeks using crutches, progressing to partial weight bearing at 4 weeks and full weight bearing at 12 weeks. Progression from crutches to canes.	Twice each day with assistance in the beginning.

Ergonomic Advice

The patient was instructed to avoid cross-sitting type of activities to avoid sartorius overload; increase the number of repetitions and holds for strengthening exercises and progression with weight; avoid high-contact sports or activities and use comfortable furniture and western toilet utility.

Outcome measures

The following outcome measures were recorded post-rehabilitation in a span of two months (Table 4).

**Table 4:** Outcome measures recorded for the patient Outcome measures on the day of surgery, the day of discharge, and the day of follow-up.

Sr.no	Outcome Measures	On the day of surgery	On the day of discharge	On the day of follow-up
1.	Range of motion	Hip flexion	0°-20°	0°-70°
		Knee flexion	0°-40°	0°-60°
2.	Functional Independence Measure (FIM)	3/7	5/7	6/7
3.	Lower Extremity Functional Scale (LEFS)	5/80	38/80	60/80

## Discussion

A fixed hip flexion deformity following retroperitoneal tumour excision is a common complication. Due to a lack of awareness and the facility of physical therapy, a large population is unaware of its significance in post-surgical incidences. Additionally, severe lumbar hyperlordosis, pelvic anteversion, and even a horizontal sacrum can result from fixed hip flexion [6]. The present case is of a 32-year-old woman who has had a chief complaint of pain in her right hip for one year. She has a history of retroperitoneal tumour excision three years ago, which was followed by difficulty walking, sitting, etc. The patient had a history of forward bending while walking. Additionally, she also complained of pain in her back. Upon X-ray investigations and clinical assessment, it was found to be functional scoliosis that developed secondary to the hip flexion deformity as diagnosed. Peterson S, in his report of a 72-year-old woman post-iliopsoas tenotomy, highlights the significance of intensive physical therapy to resolve complications, improve joint range of motion, and improve functionality [7]. van Bosse et al., through their study, mention gait deviations and explain the importance of gait rehabilitation post-lower extremity contracture or deformity [8]. In her study, Shimada T concludes the significance of regulating the position of the trunk and hips along with spine alignment through postural correction [9]. Pantzar-Castilla et al. explain the utility of physiotherapy for functional mobility, standing, and transfer ability in post-flexion contracture surgical cases [10]. Xavier et al. summarised that the respiratory aerobic training programme improved several respiratory parameters in patients with non-structural scoliosis [11]. The management provided to our patient covers goals from respiratory to gait training aspects for the patient to get back to a near-preoperative state and have more than just optimal functioning. Nicodemo et al. concluded that physical therapy is highly important for maintaining postoperative hip mobility, enhancing muscular performance, and if at all feasible, restoring walking capacity [6]. Passive prolonged stretching proves to be more successful with better results as it alters physiologic responses when treating flexion contracture.

## Conclusions

An intensive physiotherapy training protocol was planned for our patient over the course of two months and improvement was recorded positively, as depicted in the outcome measures. An effective approach and detailed attention to avoid complications helped our patient achieve her personal goals, enhanced confidence, and reduced dependency.
